# Vick's Floral Guide for 1874

**Published:** 1873-12

**Authors:** 


*Vick's Floral Guide.* Published by James Vick, Florist, Rochester New York.

The number for 1874, has been received, and as usual is beautifully embellished, the frontispiece being a magnificent colored plate of a double Portulaca. This useful guide is published quarterly at the low price of 25 cents a year, the whole number containing 200 pages, 500 engravings and a colored plate, and printed in English and German. Mr. Vick is one of the most enterprising and liberal gentlemen in his profession, and the quality of the seeds he sends out every year cannot be surpassed. He truly merits the success which has attended his efforts.

